# Cardiac Autonomic Function and Psychological Characteristics of Heterosexual Female Perpetrators of Intimate Partner Physical Aggression

**DOI:** 10.1177/0886260518775748

**Published:** 2018-05-28

**Authors:** Artur Brzozowski, Steven M. Gillespie, Louise Dixon, Ian J. Mitchell

**Affiliations:** 1 University of Birmingham, UK; 2 University of Liverpool, UK; 3 Victoria University of Wellington, New Zealand

**Keywords:** resting heart rate, heart rate variability, intimate partner violence, aggression, psychopathy

## Abstract

Intimate partner violence is predominantly viewed as a social problem of men’s violence against women. However, a growing evidence base suggests an equal prevalence rate for male and female perpetrated intimate partner physical aggression. Moreover, female perpetrated intimate partner violence is often assumed to be reactive, yet there is limited evidence to support this notion. In this article, we describe the results of two studies that investigated the prevalence of female perpetrated intimate partner physical aggression, and its correlates in heterosexual female university students. The relationships of personality traits, resting heart rate, and heart rate variability (a correlate of vagal activity) were compared between females who did and did not report having engaged in intimate partner physical aggression. In Study 1, we found that 30.9% of participants reported enacting intimate partner physical aggression during the preceding 12 months. This finding suggests that a considerable number of undergraduate females aggress against their intimate partners. Perpetrators, relative to nonperpetrators, scored higher on secondary psychopathic traits. In Study 2, female intimate partner violence was shown to be associated with low resting heart rate and high heart rate variability. Perpetrators, relative to nonperpetrators, scored higher on psychopathic traits that index emotional resilience and unempathic tendencies, and reported increased proactive and reactive aggression. This raises the possibility that some incidences of female intimate partner physical aggression represent proactive aggressive acts. These findings also support the frequently found association between low resting heart rate and aggression, but raise the prospect that the reported aggressive acts reflect high heart rate variability and strong parasympathetic nervous system activity.

## Introduction

### Gendered Perceptions of Intimate Partner Violence (IPV)

IPV can be conceptualized as a form of physical, sexual and psychological aggression perpetrated against an intimate partner (Dixon & Graham-Kevan, 2011). Although IPV is predominantly viewed as a social problem of men’s violence against women, women are also aggressive in intimate relationships (Dixon & Graham-Kevan, 2011). Indeed, a gendered perception of IPV stands in contrast to a growing evidence base which suggests that male and female acts of violence occur at approximately equal rates (Moffitt, 2001; O’Leary, Smith Slep, & O’Leary, 2007), and that this is also true for aggressive acts in intimate relationships (Esquivel-Santoveña & Dixon, 2012). However, the psychological and psychophysiological characteristics of female IPV perpetrators remain poorly understood.

The U.K. Office for National Statistics (2016) reports that twice as many females as males aged 16 to 59 were victims of any form of IPV during the 12 months preceding the survey. This accounted for 8.2% of women and 4.0% of men being victimized. However, there were no significant sex differences in the prevalence of physical violence and injury among IPV sufferers. Similar results are reported in a meta-analysis based primarily on studies with young samples in the United States of America, which found that females physically aggress against their intimate partners slightly more often than males, although injury was inflicted at a higher rate by men (Archer, 2000). Studies with university students have also found that female perpetrated IPV rates are higher than those for men (Straus, 2008). It is therefore important to progress the understanding of female intimate partner aggression.

### Differentiating Partner Violent and Nonpartner Violent Individuals

Partner violent individuals have been differentiated from nonpartner violent individuals in terms of their personality traits. This holds true for male and female perpetrators. For example, females arrested for IPV have elevated levels of histrionic, narcissistic and compulsive personality traits measured with the Millon Clinical Multiaxial Inventory–III (MCMI-III; Simmons, Lehmann, Cobb, & Fowler, 2005), increased impulsivity (Caetano, Vaeth, & Ramisetty-Mikler, 2008), and elevated features of antisocial (Goldenson, Geffner, Foster, & Clipson, 2007), and borderline personality (Henning, Jones, & Holdford, 2003). Psychopathic and antisocial tendencies and borderline features are also of particular importance among male perpetrators, and have been successfully used in the development of well validated typologies of intimate partner aggressors (Holtzworth-Munroe, Meehan, Herron, Rehman, & Stuart, 2000). The presence of these subtypes highlights the importance of examining theoretically important dimensions in partner violent individuals.

Psychopathic traits, such as empathy deficits and impulsivity, have been shown to exist on a continuum and are measurable in both forensic and nonforensic samples (Marcus, John, & Edens, 2004). Certain psychopathic traits have been linked with both premeditated and impulsive aggression (Cornell et al., 1996; Patrick, Fowles, & Krueger, 2009). For example, *meanness*, characterized by callousness and a lack of empathy, is more closely related to premeditated aggression, whereas *disinhibition*, characterized by risk taking and impulsivity, is more often linked with impulsive aggression (Patrick et al., 2009). Features of borderline personality disorder (BPD) include anxiety, emotional dysregulation and high levels of impulsivity, and individuals with these features are more likely to display acts of impulsive aggression (Lieb, Zanarini, Schmahl, Linehan, & Bohus, 2004).

### Resting Heart Rate and Aggressive Antisocial Behaviors

A few studies have investigated the relationship of cardiovascular activity to IPV perpetration. For example, male to female physical aggression has been linked with low resting heart rate (rHR) (Babcock, Green, Webb, & Graham, 2004), and this relationship appears to be most pronounced for severe, rather than low level, IPV (Babcock, Green, Webb, & Yerington, 2005). More severe perpetrators also showed weaker heart rate acceleration to marital conflict discussion compared with nonabusers (Babcock et al., 2005). These findings highlight that IPV may be related to differences in cardiovascular functioning, as well as to features of BPD and psychopathic traits.

A relationship of IPV with low rHR is perhaps unsurprising when considering that a putative relationship between low rHR and aggressive/antisocial behavior has been postulated for some time (Portnoy & Farrington, 2015). The strength of this association was emphatically supported by a Swedish longitudinal study that followed more than 700,000 men for 35 years (Latvala, Kuja-Halkola, Almqvist, Larsson, & Lichtenstein, 2015), finding a quasi-linear relationship of rHR with many types of offending. This relationship was true for both violent and nonviolent crimes, and nonintentional injuries, but not sex offenses (Latvala et al., 2015). Although most of the work looking at rHR and offending has been conducted in male participants, low rHR also predicts aggressive acts in male and female antisocial youths (Ortiz & Raine, 2004).

One popular model to account for the relationship between low rHR and aggression has emphasized that the underaroused state may be aversive and consequently drives affected individuals to partake in risky activities, including antisocial behaviors, to increase autonomic arousal (Scarpa, Raine, Venables, & Mednick, 1997; Raine, 1996; Raine, 2002; Raine & Venables, 1984). Another model posits that low rHR may reflect a lack of relative fear responses to minor laboratory stressors. However, support for either position remains weak (Gillespie, Brzozowski, & Mitchell, 2018), and a recent meta-analysis of studies disputed the latter explanation (Portnoy & Farrington, 2015).

### Functioning of the Autonomic Nervous System (ANS)

A better understanding of the mechanisms underlying this robust link between low rHR and aggressive behavior may be uncovered with considering the relationship between rHR and the functioning of the ANS (Gillespie et al., 2018). The heart rate at rest is largely dictated by the balance of activity of the two branches of the ANS acting on the sinoatrial (SA) node. The sympathetic noradrenaline based division speeds up the beating of the heart while the acetylcholine based parasympathetic division slows it down. Raine (2002) has theorized that in aggressive individuals low rHR can reflect either withdrawal of sympathetic activity, or elevated vagally mediated parasympathetic activity. However, which of the two autonomic branches plays the crucial role remains open to interpretation.

Low rHR typically stems from high activity in the vagus nerve of the parasympathetic nervous system and this in turn is reflected in high resting heart rate variability (HRV). HRV refers to the subtle changes in the interbeat intervals of the heart resulting from increased activation of the vagus nerve when breathing out, a phenomenon known as the respiratory sinus arrhythmia (RSA). This increased vagal activity also exerts an effect upon brain function, and is associated with faster, and more accurate responses on tests of executive function and a greater capacity for self-regulation (Thayer, Hansen, Saus-Rose, & Johnsen, 2009). Low rHR is typically associated with high resting HRV (Task Force of the European Society of Cardiology, 1996), implying that low rHR is predictive of greater activity of prefrontal cortical areas and good control over emotional states. Although this conclusion is counterintuitive given the strong relationship between low rHR and aggressive and antisocial behavior, low rHR might relate more strongly to aggression that is predetermined and well planned, rather than affectively driven in response to a threat or provocation. Thus, for this reason it is important when distinguishing between groups of partner violent and nonpartner violent females to examine differences in the use of reactive and proactive aggression (Cima, Raine, Meesters, & Popma, 2013; Polman, de Castro, Koops, van Boxtel, & Merk, 2007).

### Reactive and Proactive Types of Aggression

Proactive aggression can emerge without any obvious antecedent, typically requires the presence of a plan, and is associated with usual capacities for executive function (Ellis, Weiss, & Lochman, 2009; Stanford, Houston, Villemarette-Pittman, & Greve, 2003). This type of aggression is associated with psychopathic traits that index empathic deficits, as well as with low rHR and high HRV (Hansen, Johnsen, Thornton, Waage, & Thayer, 2007). On the contrary, reactive aggression results from the interpretation of another’s intentions as being hostile and provocative (Dodge et al., 2015). In contrast to proactive aggression, reactive aggression is associated with impaired executive functioning (Ellis et al., 2009) and reduced HRV (Scarpa, Haden, & Tanaka, 2010). Based on these findings, Gillespie et al. (2018) propose a model of aggression whereby low rHR is associated with high HRV, and in turn with elevated capacities for executive function and self-regulation. This suggests that low rHR is not associated with a pattern of impulsive sensation seeking, but rather a more calculated and instrumental pattern of behavior.

### The Present Study

Although several studies have examined the personality traits and pattern of cardiovascular activity associated with aggression and antisocial behavior, a more limited body of work has examined these relationships in relation to IPV. An even more limited body of work has focused on female perpetrators of IPV. Thus, the aim of the present study was to investigate the prevalence of female IPV in a heterosexual university sample, as well as the associated profile of personality traits and cardiovascular activity that characterizes female intimate partner aggression. Given existing research on the nature of the relationship between rHR, HRV, and physical aggression, we focused on the prevalence of IPV physical aggression (IPV-PA), and psychological and cardiovascular correlates of this. This decision was informed by evidence that while rHR predicts violence and unintentional injuries, relationships with other types of antisocial behavior, including sexual coercion, are less clear (Latvala et al., 2015). In Study 1, we used an online survey methodology to examine the prevalence of IPV-PA in a university sample, and compared differences between IPV physical aggressors and nonaggressors on relevant personality traits, including psychopathy, borderline personality, and anxiety. In Study 2, we compared IPV physical aggressors and nonaggressors on patterns of cardiovascular activity and personality traits recorded during a lab-based study. To test the hypothesis that low rHR and high HRV designates a particular profile at increased risk for proactive aggression, but not reactive aggression, we examined the interrelationships of rHR, HRV, and reactive and proactive aggressive traits.

We predicted that females who have engaged in IPV-PA would show a low rHR and increased levels of HRV compared with those who have not engaged in IPV-PA. Given that positive relationships exist between HRV, executive functioning, and emotion regulation, we predicted that those who had perpetrated IPV-PA would also show elevated levels of psychopathic tendencies that index emotional resilience and a lack of empathy, but not elevated features of BPD. We also predicted that low rHR and high HRV would be associated with increased proactive aggression, but not reactive aggression, consistent with the model outlined by Gillespie et al. (2018).

## Method

Two separate studies were conducted, and both recruited only female university students as participants. An overwhelming majority of the participants reported being heterosexually orientated. As IPV appears to be more prevalent in nonheterosexual samples (Whitton, Newcomb, Messinger, Byck, & Mustanski, 2019), nonheterosexual females were excluded from both studies. The first was an online study that examined the prevalence of female perpetrated IPV-PA, and the relationships between female perpetrated IPV-PA and a series of personality traits. The second study was a laboratory-based exploration of the relationship between female perpetrated IPV-PA and resting cardiovascular activity. Ethical approval for both studies was granted by the University of Birmingham Committee for Ethical Review. Participants signed their fully informed consent and received either course credit or a monetary reward of 10 pounds for their participation.

### Study 1

#### Participants

A sample of 443 heterosexual female students, aged 18 to 45 (*M* = 19.37, *SD* = 1.94) were recruited from a U.K. based university via the local research participation scheme. The participants were mainly White (73%), British (89%), and in a stable relationship (64%).

#### Measures

##### Conflict Tactics Scale–2 (CTS2)

The CTS2 is a 78-item questionnaire that measures prevalence and chronicity of IPV perpetration and victimization. The CTS2 includes 5 subscales, namely Negotiation, Psychological Aggression, Physical Assault, Aexual Coercion, and Injury. Items on all but the negotiation scale are classified as either minor or severe actions. For example, “I slapped my partner” comprises a minor assault, whereas “I beat up my partner” is classed as a severe assault. Although the CTS2 can be used as a dimensional measure of the chronicity of IPV, the current study used a dichotomous approach that is consistent with other studies that aimed to examine differences between perpetrators and nonperpetrators (Theobald, Farrington, Coid, & Piquero, 2016). This approach had an additional advantage of avoiding issues with scale reliability in community samples where some of the more severe behaviors are completely absent (Straus, 2004), and in female community samples where reliability estimates for individual subscales have been reported as low as .18 (Yun, 2010). Moreover, the current study used the physical assault subscale to discriminate between perpetrators and nonperpetrators. This is because the link between low rHR and violent offending is well evidenced whereas relationships with other types of antisocial behavior, including sexual coercion, are less consistently reported (Latvala et al., 2015).

##### Levenson Self Report Psychopathy scale (LSRP)

The LSRP inventory is intended for use in assessing psychopathic personality traits in nonoffending samples. It consists of two subscales, the first is a 16-item scale for assessing primary psychopathic traits, while the second is a 10-item scale for assessing secondary psychopathic traits. The primary subscale (LSRP-I) focuses on the selfish and uncaring characteristics associated with Factor 1 of the Psychopathy Checklist–Revised (PCL-R) (Hare, 2003), and the secondary subscale (LSRP-II) assessed the behavioral and lifestyle factors associated with PCL-R Factor 2, such as boredom and impulsivity. Good internal validity has been reported for the LSRP with a Cronbach’s alpha of α = .82 for the primary subscale and α = .63 for the secondary subscale (Levenson, Kiehl, & Fitzpatrick, 1995). Values for the current study are α = .85 and α = .66 for the primary and secondary scales, respectively.

##### State Trait Anxiety Inventory (STAI)

The STAI is a 40-item inventory with 20 items assessing trait anxiety (STAI-T) and 20 for state anxiety (STAI-S). Trait anxiety reflects anxiety as a personality trait whereas state anxiety refers to transient feelings of anxiety. In the current study, only trait anxiety was assessed as we were interested in the more enduring trait characteristics. Participants rate the extent to which each item applies to them on a 4-point Likert-type scale. Reported Cronbach’s alpha scores for the STAI are in the range of α = .86 to α = .95 (Spielberger, Gorsuch, Lushene, Vagg, & Jacobs, 1983). Here, reliability values for trait anxiety reached α = .82.

##### MCMI-III

The MCMI-III (Millon, Millon, Davis, & Grossman, 2009) is a comprehensive personality inventory containing 27 scales. The borderline scale includes 16 statements to which the respondent can either agree or disagree. This measure was devised to assess traits in relation to the *Diagnostic and Statistical Manual of Mental Disorders* (4th ed., text rev.; *DSM*-*IV-TR*; American Psychiatric Association, 2000) criteria for the diagnosis of BPD. Cronbach’s alpha for the borderline scale reported by Rossi, van der Ark, and Sloore (2007) is α = .82. In the current study, reliability reached the value of α = .78.

##### Procedure

Questionnaires were uploaded and completed through an online survey hosted by the local research participation scheme. Participants received on-screen instructions and informed consent was required before demographic information was requested, including the participants’ age, nationality, ethnicity, and cohabiting status. The questionnaires were then completed in a set order: CTS2, LSRP, STAI, MCMI-III borderline features. After completion, a debrief section was displayed and the participant was granted credit.

##### Statistical analysis

IPV-PA prevalence rates in the entire sample (*N* = 443) were calculated as the percentage of participants who responded positively to at least one item on the physical assault scale of the CTS2. The CTS2 generates a lot of data about the distinct types of IPV. However, this article has focused on IPV-PA. Accordingly, we abstracted whether the individuals had perpetrated a violent act or not, splitting the entire sample in to two groups. Participants who reported physical assault against partners that occurred within 12 months of participation in the study formed one group, and participants who did not report violence formed another group. The results of Shapiro–Wilk tests in the current sample indicated that the distribution of all continuous variables significantly deviated from normality. These findings are consistent with findings from the general population indicating that distributions for psychopathic tendencies (Coid & Yang, 2008) and borderline features (Livesley, Jackson, & Schroeder, 1992) deviate from normality, with skew typically reported toward lower scores. Therefore, a series of nonparametric Mann–Whitney *U* tests for independent samples were used to compare scores on the LSRP-I and LSRP-II, STAI-T, and MCMI-III borderline features between IPV-PA perpetrators and nonperpetrators. To control for multiple testing the obtained asymptotic alpha values were corrected using the false discovery rate (FDR) procedure proposed by Benjamin and Hochberg (1995). Both the asymptotic and the corrected alpha values are reported. Approximate effect sizes (*r*) were calculated using a procedure described by Field (2013), with the following recommended norms for interpretation: small = .1; medium = .3; large = .5.

## Results

### Characterization of the Sample

Study 1 aimed to examine differences in selected personality traits between IPV and non-IPV perpetrators of physical violence in a female university sample. The percentage of participants who reported IPV-PA perpetration were similar to those previously reported in a female sample. Thus, out of 443 heterosexual female university students, 137 (30.9%) reported acts of physical aggression against their intimate partners in the previous 12 months. This proportion is similar to that reported in an equivalent USA study where the prevalence score for female IPV-PA perpetration was 35% (Straus, Hamby, Boney-McCoy, & Sugarman, 1996). Many of the aggressive acts were classed as minor although a small number of severe acts were also reported, including the use of a knife or a gun by one of the participants. Table 1 contains the means, standard deviations, medians, and the corresponding ranges for scores on the LSRP-I and LSRP-II, STAI-T, and the MCMI-III borderline scale in Study 1. These descriptive statistics are presented for IPV and non-IPV perpetrators separately. For the whole sample, (i.e., IPV and non-IPV) primary psychopathy (*M =* 28.84, *SD =* 7.02, *Mdn* = 28, *range =* 16 – 53) and secondary psychopathy (*M* = 20.85, *SD* = 4.23, *Mdn* = 21, *range* = 10-32) scores were similar to normative values observed in undergraduate females (Lilienfeld & Hess, 2001). However, the current sample showed elevated levels of trait anxiety (*M* = 45.04, *SD* = 8.97, *Mdn* = 45, *range* = 21-78) and less noticeable borderline features (*M =* 7.14, *SD =* 5.26, *Mdn* = 6, *range* = 0-24) compared with female norms for anxiety (*M* = 40.54, *SD* = 12.86) (Spielberger et al., 1983), and borderline features (*M* = 13) (Rossi et al., 2007).

**Table 1.** Descriptive Statistics and Outcomes of the Mann–Whitney *U* Tests for Variables Used in Study 1.

	IPV				Non-IPV				Mann–Whitney *U*				
	*M*	*SD*	Median	Range	*M*	*SD*	Median	Range	*U*	*z*	*p*	FDR	*r*
Primary psychopathy	29.78	7.38	29	17-53	28.42	6.82	28	16-48	18,924.5	−1.637	.102	.204	−.08
Secondary psychopathy	21.68	4.07	22	10-32	20.48	4.25	20	10-32	17,433.5	−2.839	.005	.020*	−.14
Trait anxiety	45.58	7.87	46	26-70	44.80	9.43	45	21-78	19,691.5	−1.020	.308	.308	−.05
Borderline score	7.48	5.04	7	0-24	6.98	5.36	6	0-22	19,302	−1.335	.182	.242	−.06

*Note.* IPV = intimate partner violence; FDR = false discovery rate correction; *r* = effect size.

* Significant difference IPV vs. non-IPV (FDR < .05).

### Differences in Personality Traits Between IPV and Non-IPV Perpetrators

Table 1 contains the results of the Mann–Whitney *U* tests, the corresponding FDR corrections, and the effect sizes for analyses performed in Study 1. Participants who aggressed against their intimate partners within the last 12 months had significantly higher secondary psychopathy scores compared with those who did not aggress. The two groups did not differ in primary psychopathic traits, trait anxiety, or borderline.

### Study 2

#### Participants

A sample of 92 heterosexual female university students aged 18 to 28 (*M* = 19.09, *SD* = 1.25) were recruited via the local participation scheme. Similar to the sample obtained in Study 1, participants in the current study were mainly White (95%), British (98%), and all in stable relationship.

#### Measures

The CTS2 was used as described in Study 1. The STAI and the MCMI-III were redundant because in Study 1 there were no significant links between these two scales and female IPV. The LSRP was replaced with the Triarchic Psychopathy Measure (TriPM; Patrick et al., 2009). The TriPM assesses psychopathic traits along three dimensions that are commonly referred to in the psychopathy literature (see description of measure below) and therefore helps to provide a more nuanced understanding of the differences in psychopathic traits between groups, including in terms of emotional resilience. An additional inventory was also used, namely the Reactive–Proactive Aggression Questionnaire (RPQ) to measure the planful versus impulsive nature of aggression used by participants (Raine et al., 2006).

#### Triarchic Psychopathy Measure (TriPM)

The TriPM (Patrick et al., 2009) is a 58-item instrument with three subscales. Clinically rated and self-report measures of psychopathic tendencies in adults and children typically reveal a three-factor structure, including an affective dimension that indexes impaired empathic functioning and a lack of remorse or guilt, a fearless-dominance or interpersonal dimension that indexes social dominance and emotional resilience, and an antisocial dimension that indexes impulse control problems (Patrick et al., 2009). These dimensions are indexed in the TriPM through the meanness, boldness, and disinhibition subscales, respectively. The authors report good internal consistency for the subscales ranging from α = .80 to α = .83 and good construct validity of α = .85 (Sica et al., 2015; Stanley, Wygant, & Sellbom, 2013). The consistency values for current study range from α = .77 to α = .84.

#### RPQ

The RPQ (Raine et al., 2006) consists of 23 items measuring reactive and proactive aggression. The two types of aggression differ in terms of their motivational drive. The former occurs in response to perceived provocation or threat, whereas the latter is goal orientated and premeditated. The author reports internal reliabilities for these scales as α = .86 and α = .84, respectively (Raine et al., 2006). Reliability values for the current study are α = .86 and α = .67, respectively.

#### Procedure

Participants were tested individually in a quiet research testing cubicle. After informed consent was obtained and demographic information collected the personality inventories and the CTS2 were completed. Shortly after completion of the self-report scales a silver/silver-chloride electrode was placed on each wrist (Porto & Junqueira, 2009; Russoniello, Pougtachev, Zhirnov, & Mahar, 2010) of the participants. Prior to the positioning of the electrodes the skin was prepared by gentle rubbing with an abrasive skin cream and then wiped with alcohol. A 5-min electrocardiogram (ECG) trace was recorded using a Biocom 4,000 USB device (Biocom Technologies, Poulsbo, WA) with a sampling rate of up to 1024 Hz. Participants were asked to sit still, rest hands on their thighs and keep their eyes open. On some occasions problems with conductivity across the electrodes meant that a reliable trace was not obtained. Of the 92 participants, six did not generate a successful trace.

We used the Heart Rhythm Scanner Professional Edition (Biocom Technologies, Poulsbo, WA) software to automatically compute all measures of cardiovascular activity. Resting heart rate and vagally mediated parasympathetic activity was calculated from the 5-min long epoch. The activity of the parasympathetic system is expressed using a measure of HRV. This is based on the standard deviation of successive interbeat intervals (SDNN), a reliable and widely accepted measure of HRV (Task Force of the European Society of Cardiology, 1996).

#### Statistical analysis

Similar to Study 1, and consistent with findings from the general population, examination of the data confirmed that distributions were skewed toward lower values. Therefore, nonparametric tests were selected to compare IPV aggressors and nonaggressors on psychological characteristics, and resting cardiovascular activity. The analysis, excluding partial correlations, was performed in IBM SPSS version 24. Nonparametric partial correlations were performed using Statistical Analysis Software (SAS 9.4) (SAS Institute Inc., Cary, NC, USA).

To investigate the differences between IPV perpetrators and nonperpetrators the entire sample was split into two groups. Participants who reported at least one assault against their partner within 12 months of participation in the study formed one group, and those who did not report an assault formed the second group. Differences in cardiovascular activity (rHR, HRV [SDNN]), self-report aggression (reactive, proactive) and psychopathic traits (boldness, meanness, disinhibition) were analyzed with separate Mann–Whitney *U* tests. An FDR correction (Benjamin & Hochberg, 1995) was applied to all *U* tests. Effect sizes were estimated with procedures briefly described in Study 1.

In a subsequent analysis, the two groups were merged and partial correlations of proactive and reactive aggression with HR and HRV (SDNN) were performed. Reactive and proactive aggression were interchangeably entered as a correlated or control variable to control for the typically medium sized correlations between these two constructs. Thus, the results reported here refer to the unique relationships with reactive and proactive aggression independent of the other.

## Results

### Characterization of the Sample

Study 2 aimed to expand on the findings of Study 1 by investigating the concomitants of female perpetrated partner aggression, selected cardiovascular parameters, and personality characteristics. Out of the whole sample, 37 participants (40.2%) reported IPV-PA. The aggressive acts were typically described as being mild assaults where there were no obvious lasting effects. However, five of the participants caused bruising, a sprain or small cut to their partners. A further two participants reported causing pain that lasted for 24 hr. Table 2 contains the means, standard deviations, medians, and the corresponding ranges for cardiovascular parameters and personality inventory scores used in Study 2. These descriptive statistics are presented for IPV and non-IPV perpetrators separately. For the whole sample (i.e., IPV and non-IPV) mean rHR (*M* = 79.36, *SD* = 11.44, *Mdn* = 77.7, *range* = 61-118) was comparable with normative values (*M* = 80 bpm). SDNN values (*M* = 62.86, *SD* = 29.59, *Mdn* = 55, *range* = 20-169) were higher than young female norms (SDNN: *M* = 48.7, *SD* = 19) as reported by Voss, Schroeder, Heitmann, Peters, and Perz (2015). Proactive aggression (*M* = .79, *SD* = 1.52, *Mdn* = 0, *range* = 0 – 10) and reactive aggression (*M* = 6.04, *SD* = 3.97, *Mdn* = 6, *range* = 0-22) values were slightly lower than those previously reported for a community sample of adolescent females (Fossati, Borroni, Eisenberg, & Maffei, 2010). The mean scores for boldness (*M* = 26.33, *SD* = 7.47, *Mdn* = 27, *range* = 7-44), meanness (*M* = 8.61, *SD* = 6.29, *Mdn* = 7, *range* = 0-37), and disinhibition (*M* = 14.09, *SD* = 6.94, *Mdn* = 13, *range* = 2-46) were relatively similar to normative values (Poy, Segarra, Esteller, López, & Moltó, 2014).

**Table 2.** Descriptive Statistics and Outcomes of the Mann–Whitney *U* Tests for Variables Used in Study 2.

	IPV				Non-IPV				Mann–Whitney *U*				
	*M*	*SD*	Median	Range	*M*	*SD*	Median	Range	*U*	*z*	*p*	FDR	*r*
Cardiovascular variables													
rHR	75.43	9.50	75.1	61-100	82.06	11.95	82.1	61-118	1210	−2.747	.006	.011*	−.30
HRV (SDNN)	77.39	34.86	62.9	25-169	52.88	20.31	48.5	20-100	1811	−3.582	.001	.002**	−.39
Personality variables													
Proactive Agg.	1.32	1.94	1	0-10	0.44	1.01	0	0-5	2199.5	−3.334	.001	.002**	−.35
Reactive Agg.	7.41	3.75	7	0-19	5.13	3.89	5	0-22	2154.5	−3.223	.001	.046*	−.34
Boldness	28.30	8.36	28	7-42	25.00	6.55	26	10-44	2300.5	−2.049	.040	.047*	−.21
Meanness	10.89	8.00	9	0-37	7.07	4.24	7	0-17	2249.5	−2.459	.014	.019*	−.25
Disinhibition	15.68	7.73	15	2-46	13.02	6.20	12	2-34	2325.5	−1.851	.064	.064	−.19

*Note.* Psychometric data are presented for *N* = 92 (IPV perpetrators: *N* = 37, nonperpetrators: *N* = 55), 86 of whom generated cardiovascular data (IPV perpetrators: *N* = 35, nonperpetrators: *N* = 51). IPV = intimate partner violence; FDR = false discovery rate correction; *r* = effect size; rHR = resting heart rate; HRV (SDNN) = resting heart rate variability expressed in standard deviation of successive interbeat intervals.

* Significant difference IPV vs non-IPV (FDR < .05). **Significant difference IPV vs. non-IPV (FDR < .01).

### Differences Between IPV and Non-IPV Groups

Table 2 contains the results of the Mann–Whitney *U* tests, the corresponding FDR corrections, and the effect sizes for analyses performed in Study 2. The Mann Whitney *U* tests examining differences in rHR and HRV (SDNN) revealed that perpetrators showed a lower rHR and higher SDNN values compared with nonperpetrators. Two more Mann–Whitney *U* tests were conducted to test whether IPV-PA perpetrators scored higher on self-report aggression than nonperpetrators. The outcomes revealed significantly higher levels of both proactive and reactive aggression among IPV-PA perpetrators. Moreover, it was hypothesized that IPV-PA perpetrators would have increased levels of psychopathic traits, mainly those that index emotional resilience and callous unempathic features. Results of the Mann–Whitney *U* tests indicated that the IPV-PA group showed significantly higher scores for boldness, and meanness, but not disinhibition.

### Partial Correlations for Cardiovascular Variables, Reactive, and Proactive Aggression

The correlation between rHR and proactive aggression was not significant (*r*_s_ = −.205, *p* = .061) when reactive aggression was used as a covariate. Similarly, the correlation between rHR and reactive aggression was also nonsignificant (*r*_s_ = .039, *p* = .723) when controlling for proactive aggression.

We did, however, find a significant partial correlation between HRV (SDNN) and proactive aggression (*r*_s_ = .294, *p* = .006) when reactive aggression was used as a covariate. There was no significant relationship between HRV (SDNN) and reactive aggression (*r*_s_ = .057, *p* = .602) when controlling for proactive aggression. Thus, proactive, but not reactive, aggression was associated with higher levels of HRV (SDNN).

## Discussion

This study aimed to establish the prevalence of IPV-PA in a university sample, and to investigate the relationship of female perpetrated IPV-PA with relevant personality traits and cardiac autonomic function. The results of Study 1 showed that about a third of heterosexual female participants (30.9%) physically aggressed against their intimate partners in the previous 12 months. These finding were further supported by results from Study 2, which showed that 40.2% of females had aggressed against their partner. Though contrary to some models of IPV that emphasize patriarchy as a motivating factor for this type of aggression, the findings are in keeping with several recent studies, including a meta-analysis by Williams, Ghandour, and Kub (2008), which suggest that IPV is not solely an issue of men’s violence against women.

Consistent with our hypothesis, results from Study 2 showed that female IPV-PA perpetrators had a lower rHR than nonperpetrators. This finding is consistent with the results of an earlier study of male IPV perpetrators which showed that those with a lower rHR committed more severe acts of IPV than those with a higher rHR (Babcock et al., 2005). This observation is also supported by the well-established finding that low rHR in males is related to aggressive behavior and several forms of offending, as well as unintentional injuries (Latvala et al., 2015). These similarities between findings suggest that the expression of aggressive tendencies may originate by the same physiological mechanisms for males and females. This interpretation is in keeping with the results of a meta-analysis that reported low rHR in male and female samples of antisocial adolescents (Ortiz & Raine, 2004).

Although the relationship of rHR with aggression is emphatically supported (Latvala et al., 2015), the mechanisms underlying this relationship have been extensively debated (Raine, 2002; Wilson & Scarpa, 2014). A common interpretation of the relationship is that low rHR reflects low autonomic arousal resulting in impulsive sensation seeking, and aggressive behaviors (Raine, 2002). However, this explanation has received only limited support. It has been suggested that this debate can be better informed by a consideration of the separable influences of the parasympathetic and the sympathetic divisions of the ANS on the resting rate of the heart (Gillespie et al., 2018). Consistent with an inverse relationship of rHR with HRV, in Study 2, we found that females who had engaged in IPV-PA showed higher levels of SDNN, indicative of greater vagally mediated HRV, compared with those who had not engaged in IPV-PA. Furthermore, we found a significant positive relationship of SDNN values with proactive, but not reactive, aggression. Extensive lines of experimental evidence have indicated that HRV is associated with increased executive functioning capacities and good emotion regulation (Thayer, Åhs, Fredrikson, Sollers, & Wager, 2012). These qualities may reflect the finding that the connectivity between the dorsolateral prefrontal, anterior cingulate, limbic, and brainstem structures is heightened during states of elevated HRV (Chang et al., 2013). Although we did not measure executive function in the present study, when taken together these findings suggest that instances of female perpetrated IPV may reflect more planful, instrumental acts of aggression, rather than affectively driven reactions to provocation. These results are consistent with the model outlined by Gillespie et al. (2018) suggesting that low rHR and associated increases in HRV may contribute to a pattern of calculated risk-taking that underlies proactive, but not reactive aggression.

The suggestion that female IPV-PA may reflect more instrumental motives is inconsistent with the commonly held assumption that intimate partner physical aggression is perpetrated predominantly by males, and that females are aggressive only in response to aggression from a male partner (Capaldi, Kim, & Shortt, 2004). However, Whitaker and colleagues report evidence that is contradictory to this assumption, showing that in nonreciprocally violent relationships, women were the perpetrators in more than 70% of the cases (Whitaker, Haileyesus, Swahn, & Saltzman, 2007). Thus, female perpetrated intimate partner aggression does not simply reflect a reaction to male aggression.

The proposition that instances of female perpetrated IPV can reflect instrumental acts is also supported by findings from Studies 1 and 2 that heterosexual female IPV is related to elevated secondary psychopathy scores (Study 1), as well as higher scores on the boldness and meanness subscales of the TriPM (Study 2). These psychopathic tendencies are associated with a lack of empathy, good emotional resilience, and increased use of both reactive and proactive aggression (Cima et al., 2013). There is some consistency here between our results in a female sample, and findings from male IPV samples that also found elevated psychopathic tendencies (Theobald et al., 2016). These findings from personality inventories compliment data on cardiac autonomic function, and further support the argument that the motivations for female IPV-PA may be both instrumental, as well as reactive. However, it is important to note that reactive and proactive aggression are highly correlated, and aggressive acts often reflect components of each.

A limitation of the current study is the reliance on HRV to act as an index of parasympathetic function. The measurement of HRV is influenced by mechanisms other than vagal activity, including respiration rate (Grossman & Taylor, 2007), and residual inspiratory vagal activity (Grossman & Kollai, 1993). Nonetheless, HRV (calculated as SDNN) remains a widely recognized and recommended measure of vagal influences on the rate of the heart (Task Force of the European Society of Cardiology, 1996). Although our prediction that SDNN values would be associated with proactive aggression was confirmed, contrary to our prediction, the relationship of rHR with proactive aggression was nonsignificant. This pattern of results is nonetheless consistent with those of a similar study that investigated cardiovascular activity and aggression in a sample of children (Scarpa et al., 2010). An additional limitation surrounds the method of identifying IPV-PA perpetrators. Although prevalence rates in this study are in line with existing estimates, the findings presented here are based on self-reported acts of IPV and may therefore be subject to social desirability bias. Furthermore, we only collected data from heterosexual, undergraduate female students, majority of whom described themselves as White and British. The extent to which a similar pattern of results would be found among individuals from other racial backgrounds, nationalities, and nonstudent, adult, and male samples is worthy of further investigation.

Although our findings suggest a role of the parasympathetic division of the ANS in the rHR-aggression relationship, the role of the sympathetic division remains unclear and future studies should seek to assess both parasympathetic and sympathetic influences on the heart. It is also important to examine the extent to which low rHR is a characteristic of particular subtypes of IPV perpetrators, with such results having the potential to inform clinical decision making. Although causal mechanisms cannot be inferred from the data reported here, several longitudinal studies that have taken in to account potential moderators and mediators, including parental adversity, gender, family education, and intelligence, have confirmed the relationship of low rHR with later aggression (Portnoy & Farrington, 2015). This relationship therefore appears to be highly generalizable and may also apply to males. Understanding these relationships has clinical implications for the development of effective interventions, and it is intriguing that various interventions, including educational and nutrition enrichment programs (Rocque, Welsh, & Raine, 2012), and family interventions (Raine, Mellingen, Liu, Venables, & Mednick, 2003), appear to have an effect on biological function. Future longitudinal studies should assess the impact of such programs on the incidence of IPV.

In conclusion, results presented here suggest that female IPV-PA is perpetrated at similar rates to those reported among males, with aggressors showing high levels of both reactive and proactive aggression, and elevated psychopathic tendencies indexing emotional resilience and a callous lack of empathy. Furthermore, the cardiac autonomic function of female IPV-PA perpetrators appears to be characterized by a low rHR and elevated levels of parasympathetic activity, as reflected in elevated levels of HRV. These results highlight the need to reappraise the previously hypothesized mechanisms driving the relationship between low rHR and aggressive and antisocial behavior. Previous work has emphasized the possibility that low rHR might reflect an aversive state of sympathetic under arousal, rather than elevated parasympathetic activity. Our data provide support for an alternative mechanism whereby low rHR is driven by increased vagal activity. However, these data cannot rule out a concomitant role of the sympathetic division. Previous work highlights that vagally mediated HRV is associated with benefits in executive functioning and emotion regulation, and these capacities would be expected to contribute to the planned and instrumental nature of proactive aggression (Gillespie et al., 2018).
